# Tissue factor-targeted lidamycin inhibits growth and metastasis of colon carcinoma

**DOI:** 10.3892/ol.2013.1437

**Published:** 2013-07-03

**Authors:** QING ZHANG, XIUJUN LIU, CAIHONG LI, DONGSHENG LIAO, ZHIGANG OUYANG, JUNNIAN ZHENG, XU SONG

**Affiliations:** 1 Jiangsu Key Laboratory of Biological Cancer Therapy, Xuzhou Medical College, Xuzhou, Jiangsu 221002, P.R. China; 2 Center for Functional Genomics and Bioinformatics, College of Life Science, Sichuan University, Chengdu, Sichuan 610064, P.R. China; 3 Institute of Medicinal Biotechnology, Chinese Academy of Medical Sciences and Peking Union Medical College, Beijing 100050, P.R. China; 4 The Affiliated No.2 Hospital of Xuzhou Medical College, Xuzhou, Jiangsu 221002, P.R. China

**Keywords:** lidamycin, tissue factor, factor VII, colon carcinoma, metastasis

## Abstract

Colon cancer is the third most common cancer in the world. The overexpression of tissue factor (TF) in colon cancer cells makes it an ideal target for colon cancer therapy. The purpose of the present study was to develop a TF-targeting energized fusion protein, mlFVII-LDP-AE, which is composed of a mouse Factor VII light chain (mlFVII) as the targeting domain conjugated to the highly cytotoxic antibiotic lidamycin (LDM, LDP-AE) as the effector domain. The potential efficacy of mlFVII-LDP-AE for mouse colon cancer therapy was tested in a mouse colon cancer subcutaneous xenograft model and a live metastasis model in BALB/c mice. mlFVII-LDP-AE showed a tumor growth inhibition rate of 91.2% (at a dose of 0.8 mg/kg) and a tumor metastasis inhibition rate of 84.7% (at a dose of 0.6 mg/kg). The results showed that mlFVII-LDP-AE was able to effectively inhibit the growth and metastasis of mouse colon cancer. As human TF and FVII have features similar to those of mice, human FVII light chain (hlFVII)-targeted LDM (hlFVII-LDP-AE) may be expected to have therapeutic potential for human colon cancer.

## Introduction

Colon cancer is the third most common cancer in the world. The primary treatment of colon cancer is to surgically remove part of or the whole of the colon. Chemotherapy is an important supplementary approach that is capable of prolonging survival for individuals whose cancer has spread to other organs (1,2). Targeted chemotherapy specifically kills tumor cells with minimal toxicity to normal cells.

Tissue factor (TF) is a transmembrane receptor that is overexpressed in angiogenic tumor vascular endothelial cells and numerous types of cancer cells, including solid tumors and leukemia (3). The TF levels of cancer cells are ~1,000-fold greater than those of their normal counterparts. This overexpression is also observed in clinical samples of numerous types of human cancer, with only a few exceptions (e.g. renal cancer) (4). Therefore, TF is an ideal target for cancer therapy. Factor VII (FVII) is the natural ligand of TF and binds TF with exceptionally high specificity and affinity (~10^−12^ M) (5). Therefore, FVII may be used as a targeting vehicle to develop a targeted therapeutic agent.

Several TF-targeting therapeutic agents have been tested for the treatment of cancer and non-cancerous diseases. Hu *et al* developed the first TF-targeting agent, the antibody-like FVII-targeted Icon (FVII/IgG1 Fc), for cancer immunotherapy in 1999 (6). In the following years, Icon immunotherapy was tested for the eradication of the pathological neovasculature in other tumors (7–9). Hu *et al* also developed two FVII-targeted photodynamic therapeutics for breast cancer by conjugating Sn(IV) chlorine e6 (SnCe6) (10) or verteporfin (3) to FVII. Similarly, Shoji *et al* reported the use of recombinant human FVII as a carrier for the targeted delivery of a potent synthetic curcumin analog (EF24) to TF-expressing tumor cells (11).

In order to reduce the coagulation activity of FVII-targeted therapeutics, the serine protease activity of FVII was inactivated by biological or chemical methods in the previous studies. These methods have the following drawbacks: i) The risk of the coagulation cascade reaction initiated by FVII-targeted therapeutics may not be completely excluded; ii) the production cost of the therapeutics is expensive; and iii) the high molecular weight of the therapeutics negatively affects their ability to penetrate into tumors.

Activated FVII is composed of a 20-kDa amino-terminal light chain and a 30-kDa carboxy-terminal heavy chain, which are linked by a disulfide bond (12). The light chain binds to TF and the heavy chain initiates the blood coagulation pathway (13,14). We previously demonstrated that the light chain of FVII was able to effectively bind to TF (15). In the present study, the light chain of mouse FVII (mlFVII) was selected as the targeting vehicle. The single-chain mlFVII molecule is significantly smaller than the parental two-chain FVII molecule, which should provide two therapeutic advantages: i) Facilitating access of the mlFVII-targeting therapeutics to solid tumors (16); and ii) completely preventing blood clots that may otherwise occur when the two-chained FVII molecule binds to TF (14).

The cytotoxic molecule selected was lidamycin (LDM), a member of the enediyne antibiotic family derived from *Streptomyces globisporus* C1027 (17). The LDM molecule is composed of an 843-Da enediyne chromophore (AE), which binds DNA in the minor groove and causes double-stranded DNA breaks and tumor cell death, and a 10.5-kDa apoprotein of LDM (LDP) that forms a hydrophobic pocket protecting AE (18). LDM is cytotoxic to cultured tumor cells (19).

In the present study, an energized fusion protein, mlFVII-LDP-AE, was constructed containing mlFVII as the targeting domain and LDM (LDP-AE) as the effector domain. The results of the study showed that the energized fusion protein was able to inhibit not only the growth, but also the metastasis of mouse colon cancer.

## Materials and methods

### Cell culture

The human liver cancer lines HepG2 and 7721, human lung cancer lines A549 and NCI-H292, human colorectal cancer lines HCT-116, human skin fibroblast BJ and human embryonic kidney (HEK)293 cells were cultured in Dulbecco’s modified Eagle’s medium. Human breast cancer MCF-7 cells were cultured in Eagle’s minimum essential medium. Human breast cancer MDA-MB-231 cells were cultured in Leibovitz’s L-15 medium. Cells from mouse colon cancer line C26, mouse melanoma line B16F10 and mouse prostate cancer line RM-1 were cultured in RPMI-1640 medium. All media were supplemented with 10% fetal bovine serum (FBS) when used. All media and FBS were purchased from GIBCO (Carlsbad, CA, USA).

### Construction of expression plasmid

**Figure 1:**
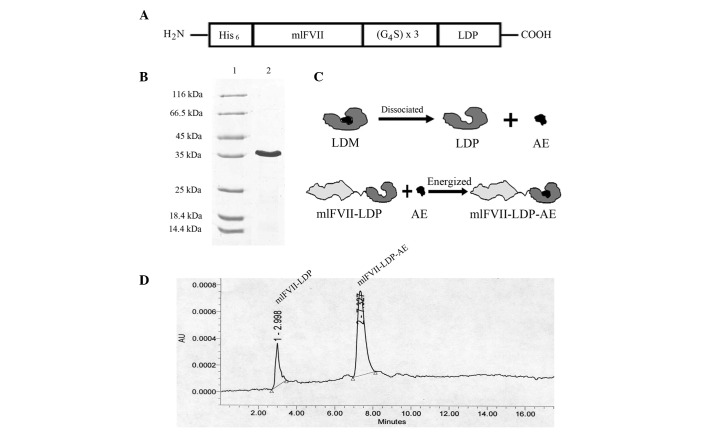
(A) Domain diagram of recombinant protein mlFVII-LDP. (B) SDS-PAGE (lane 2) detection of mlFVII-LDP. (C) Schematic representations of the dissociation of LDM into AE and LDP and the reconstitution of mlFVII-LDP-AE. (D) Analysis of the energized fusion protein, mlFVII-LDP-AE, was performed via reverse-phase HPLC. His6, purifying tag composed of six Histidines; mlFVII, light chain of mouse factor VII; (G4S)3, flexible linker composed of triplicate GGGGS; LDP, apoprotein of lidamycin; AE, chromophore of lidamycin; HPLC, high-performance liquid chromatography; AU, absorbance unit.

A diagram of the recombinant protein, mlFVII-LDP, is shown in Fig. 1A. The expression plasmid of mlFVII-LDP was constructed by conventional molecular cloning techniques. The DNA fragment encoding mlFVII (152 amino acids) was cloned from the plasmid pcDNA3.1-mFVII/hFc (6). The coding sequence for LDP was cloned from the plasmid pET-VH-LDP (18). The recombinant DNA fragment encoding the fusion protein was spliced by overlap extension PCR. mlFVII and LDP were linked by a flexible peptide. A 6xHis tag sequence was added to the N-terminal of the recombinant protein. The recombinant DNA fragment was inserted into the expression vector pET-19b (Novagen, Darmstadt, Germany).

### Expression and purification of recombinant proteins

The recombinant protein, mlFVII-LDP, was expressed and purified as described previously (15,19). The recombinant plasmid was transformed into Rosetta-gami B (DE3) pLysS competent cells (Novagen). A single colony was inoculated into 5 ml LB medium (pH 7.5) containing 100 μg/ml ampicillin, 34 μg/ml chloramphenicol, 15 μg/ml kanamycin and 12.5 μg/ml tetracycline and cultured overnight at 37ºC. The next day, 1 liter of culture medium was inoculated with the overnight culture and incubated with agitation at 37ºC until A_600_ = 0.6–1. Isopropyl-β-D-1-thiogalactopyranosid (IPTG) was added to the medium to a final concentration of 0.8 mM. Subsequent to being induced for 6 h at 37ºC, the bacteria were lysed using pulse sonication followed by 60 min centrifugation at 48,400 × g. His-tagged recombinant protein was purified by affinity chromatography (HisTrap HP; GE Healthcare, Little Chalfont, UK) and ion exchange chromatography (HiTrap Q HP; GE Healthcare) according to the manufacturer’s instructions.

### Preparation of energized fusion protein hlFVII-LDP-AE

The energized fusion protein hlFVII-LDP-AE was prepared as described previously (15). In brief, the active AE of LDM was separated using a C4 column (GE Healthcare) with a 22% acetonitrile in 0.05% trifluoroacetic acid mobile phase. The AE-containing solution was added to mlFVII-LDP/phosphate-buffered saline (PBS; 10 mM, pH 7.0) with a molecular ratio of 3:1 and was incubated at 4ºC for 12 h with rocking. Free AE was removed with a Sephadex G-75 column (GE Healthcare). The assembled energized fusion protein was confirmed by reverse-phase high-performance liquid chromatography (HPLC) using a Vydac C4 300A column (Grace, Deerfield, IL, USA). Absorbance was measured at 350 nm.

### Western blot analysis

The cells were cultured in six-well plates. When the cells were 80–90% confluent, they were washed twice with PBS. Subsequently, 1 ml ice-cold PBS was added to the wells. The cells were scraped down and collected by centrifugation at 800 × g for 5 min, then 300 μl ice-cold lysis buffer (20 mM Tris-HCl, 150 mM NaCl, 10 mM KCl, 0.5 mM EDTA, 1.5 mM MgCl_2_, 0.5 mM PMSF, 2 mM DTT, 2.5 mM CaCl_2_, 0.5% NP-40 and 10% glycerol; pH 7.9) was added and the cells were incubated on ice. The cell suspensions were vortexed five times with 2 min intervals. Lysates were centrifuged at 16,000 × g at 4ºC for 5 min. The lysate supernatants were resolved with a 12% SDS-PAGE gel and detected using a mouse anti-human TF antibody (MAB23391, R&D Systems, Minneapolis, MN, USA). β-actin was also detected as a control.

### Fluorescence-activated cell sorting (FACS) analysis

The cells (1×10^6^) were incubated with a goat anti-mouse TF antibody (AF3178; R&D Systems) in 1 ml PBS for 30 min at room temperature. The cells were washed twice with PBS and incubated with a mouse anti-goat IgG antibody conjugated to FITC (Santa Cruz Biotechnology, Inc., Santa Cruz, CA, USA; sc-53800) for 30 min at room temperature. The cells were then washed, resuspended and evaluated with a FACS machine (BD Biosciences, San Diego, CA, USA). Matched isotype control antibody was used in all analyses. For analysis, the relative log fluorescence of live cells was determined using a FACScan flow cytometer with CellQuest software (BD Biosciences).

### Tumor xenograft growth inhibition in vivo

Mouse colon cancer C26 cells in the exponential growth phase were dissociated with 5 mM EDTA in PBS, centrifuged, washed and resuspended in PBS. Subsequently, six-week-old female BALB/c mice (Institute of Laboratory Animal Sciences, Chinese Academy of Medical Sciences, Shanghai, China) were injected subcutaneously (s.c.) into the left armpit with 5×10^6^ C26 cells per mouse in 100 μl of PBS. This study was approved by the ethics committee of Sichuan University (Chengdu, China). At three weeks post-injection, tumors were aseptically dissected and sections of tumor tissue (2–6 mm^3^ in size) were transplanted s.c. into the left armpit of the mice using a trocar. After 10 days, the tumors were removed and the tumor cells were resuspended with PBS (W:V=1:5).

On day 0, the tumor cell suspension was injected s.c. into the left armpit of the mice at 200 μl per mouse. On day 3, the mice were divided into five groups (n=10). Four groups were injected i.v. into the tail vein twice at days 3 and 10 with LDM (0.05 mg/kg, tolerated dose) or mlFVII-LDP-AE (0.2, 0.4 and 0.8 mg/kg) in 200 μl PBS. The control group was injected with 200 μl PBS. On day 14, the tumors were removed and weighed. The tumor growth inhibition rate (TGIR) = 1 − tumor weight (treated)/tumor weight (control) × 100. The mice were weighed on days 3 and 14.

### Tumor metastasis inhibition in vivo

A mouse colon cancer C26 cell suspension was prepared, as described previously. On day 0, the cell suspension was inoculated into the spleens (caudal end) of the mice at 20 μl per mouse. Any bleeding was stopped using thrombin. On day 3, the mice were divided into five groups (n=10). Four of the groups were injected i.v. into the tail vein twice, at days 3 and 10, with LDM (0.05 mg/kg) or mlFVII-LDP-AE (0.2, 0.4 and 0.6 mg/kg) in 200 μl PBS. The control group was injected with 200 μl PBS. On day 14, the mice were dissected and the metastatic nodes on the liver surfaces were counted. The tumor metastatic inhibition rate (TMIR) = 1 − tumor weight (treated)/tumor weight (control) × 100. The mice were weighed on days 3 and 14.

### Statistical analysis

Data are expressed as the mean ± SD in all cases. Comparisons of the mean values between the different treatments were performed using the two-way Independent-Sample t-test with Excel 2003 software (Microsoft, Redmond, WA, USA). P<0.05 was considered to indicate a statistically significant difference.

## Results

### Construction and preparation of mlFVII-LDP-AE

Recombinant DNA encoding the protein mlFVII-LDP was cloned and inserted into pET-19b expression vectors. After induction and purification by Ni^2+^ affinity chromatography and ion exchange chromatography, the fusion protein was detected by SDS-PAGE (Fig. 1B). The energized fusion protein, mlFVII-LDP-AE, was prepared by molecular reconstitution with AE and mlFVII-LDP (Fig. 1C). Data from reverse-phase HPLC showed that the AE molecule was integrated into mlFVII-LDP successfully and the purity of hlFVII-LDP-AE was 78.6% (Fig. 1D).

### Expression of TF in human and mouse cell lines

**Figure 2:**
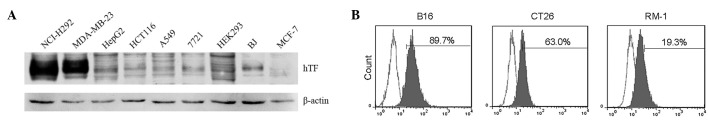
Expression of tissue factor (TF) in human and mouse cell lines. (A) Expression of TF in human cell lines analyzed by western blotting. (B) Expression of TF in mouse cancer cell lines analyzed by fluorescence-activated cell sorting (FACS).

The expression of human TF (hTF) in human cell lines was detected by western blotting. As shown in Fig. 2A, hTF has a high level of expression in the MDA-MB-231 breast cancer and NCI-H292 lung cancer cells and almost no expression in the HEK293 and MCF-7 breast cancer cells. The expression of mouse TF (mTF) was then detected in mouse cancer cell lines by FACS analysis. As shown in Fig. 2B, mTF has evidently high expression in melanoma B16F10 and colon cancer C26 cells. The mouse colon cancer C26 cell line was selected as the model cell line to study the therapeutic efficacy of mlFVII-LDP-AE for colon cancer in the present study.

### Tumor growth inhibition of mlFVII-LDP-AE in vivo

**Figure 3:**
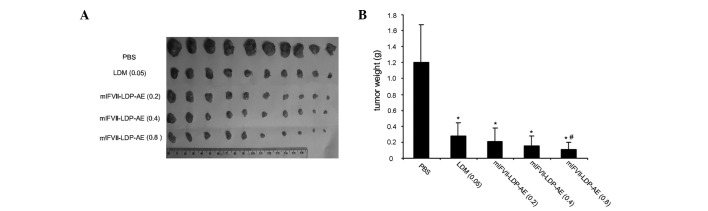
Growth inhibition by mlFVII-LDP-AE in mouse colon cancer 26 (C26) xenografts in BALB/c mice. (A) Image of the tumors removed from the mice at end of experiment. (B) Statistical results of the tumor weights. ^*^P<0.05 vs. the group treated with PBS. ^#^P<0.05 vs. the group treated with LDM. LDM, lidamycin; PBS, phosphate-buffered saline; mlFVII-LDP-AE, mouse FVII light chain (mlFVII)-targeted LDM.

The tumor growth inhibition effects of mlFVII-LDP-AE *in vivo* were tested in a BALB/c mouse xenograft model using mouse colon cancer C26 cells. At the end of the experiment, the tumors were excised (Fig. 3A) and weighed. As shown in Fig. 3B, free LDM (0.05 mg/kg, tolerated dose) showed a 77% TGIR, while mlFVII-LDP-AE at 0.2, 0.4 and 0.8 mg/kg increased the antitumor effects to a TGIR of 82.5, 87.4 and 91.2%, respectively. The TGIRs of mlFVII-LDP-AE at 0.2 and 0.4 mg/kg were not observed to be significantly different compared with that of LDM at 0.05 mg/kg (P>0.05). However, the TGIR of mlFVII-LDP-AE at 0.8 mg/kg exhibited a significant difference compared with that of LDM (P<0.05).

### Tumor metastasis inhibition of mlFVII-LDP-AE in vivo

**Figure 4:**
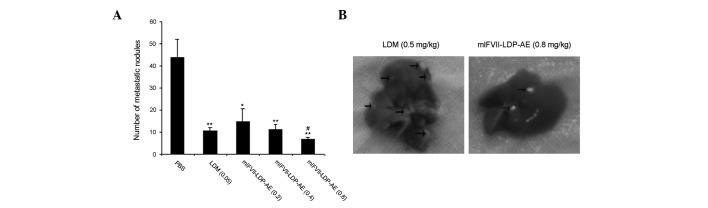
Metastasis inhibition by mlFVII-LDP-AE in mouse colon cancer 26 (C26) cells in BALB/c mice. (A) Number of metastatic nodes on liver surfaces of mice. ^*^ P<0.05 vs. the group treated with PBS. ^**^P<0.01 vs. the group treated with PBS. ^#^ P<0.05 vs. the group treated with LDM,. (B) Representative images of liver metastases in the groups treated with LDM and mlFVII-LDP-AE. Arrows, metastatic nodes. LDM, lidamycin; PBS, phosphate-buffered saline; mlFVII-LDP-AE, mouse FVII light chain (mlFVII)-targeted LDM.

The tumor metastasis inhibition effects of mlFVII-LDP-AE *in vivo* were tested with a liver metastasis C26 mouse colon cancer model in BALB/c mice. At the end of the experiment, the mice were dissected and the liver surface metastatic nodes of the mice were counted (Fig. 4B). As shown in Fig. 4A, the TMIR of free LDM (0.05 mg/kg, tolerated dose) was 76%, while that of mlFVII-LDP-AE at 0.2, 0.4 and 0.6 mg/kg was 66.9, 74.2 and 84.7%, respectively. The results of the statistical analysis showed that the TMIR of mlFVII-LDP-AE at 0.8 mg/kg was significantly different from that of LDM (P<0.05).

### Safety of mlFVII-LDP-AE for application in vivo

**Figure 5:**
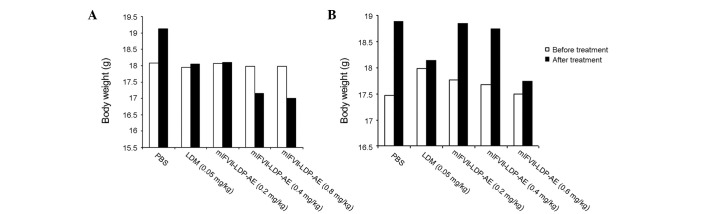
Mean body weights of mice in each group. (A) Tumor growth inhibition experiment. (B) Tumor metastasis inhibition experiment. LDM, lidamycin; PBS, phosphate-buffered saline; mlFVII-LDP-AE, mouse FVII light chain (mlFVII)-targeted LDM.

The body weight of the mice was monitored as a systemic toxicity indicator of the drug administered. In the tumor growth inhibition experiment, as shown in Fig. 5A, the body weights of the mice treated with PBS, LDM and mlFVII-LDP-AE (0.2 mg/kg) increased by 5.4, 0.6 and 2.0%, respectively at the end of the experiment. The body weights of the mice treated with mlFVII-LDP-AE at 0.4 and 0.8 mg/kg were decreased by 4.9 and 5.7%, respectively, at the end of the experiment. In the tumor metastasis inhibition experiment, the body weights of all the mice increased. The mice treated with mlFVII-LDP-AE at 0.6 mg/kg showed a 1.5% increase, while the mice treated with LDM only showed a 0.8% increase.

To summarize, the body weight loss caused by mlFVII-LDP-AE did not exceed 10%. Therefore, the dosages of mlFVII-LDP-AE used in the present study were tolerated (18).

## Discussion

Under normal conditions, TF is expressed on the extravascular cells of several normal tissues, as well as in the adventitial layer of large blood vessel walls (20,21). TF is anatomically sequestered from its natural ligand, FVII, and TF-targeting therapeutic agents circulating in blood by the intact semipermeable endothelial layer of normal blood vessels (22). Therefore, TF-targeting therapeutic agents do not damage normal tissues. In cancer patients, TF is overexpressed on angiogenic tumor vascular endothelial cells and numerous types of cancer cells. TF-targeting therapeutic agents are able to destroy tumor vessels by attacking tumor vascular endothelial cells. Systemically administered TF-targeting therapeutic agents also penetrate into tumor tissue and kill the tumor cells through leaks in the tumor vasculature (23). Therefore, TF is a safe and effective target for cancer therapy.

LDM is a member of the enediyne antibiotic family derived from *Streptomyces globisporus* C1027. LDM exhibits extremely potent cytotoxicity, antitumor activity and marked growth inhibition against transplantable tumors in mice. In terms of the half maximal inhibitory concentration (IC_50_) values, the cytotoxicity of LDM has been shown to be 10,000-fold more potent than those of mitomycin and doxorubicin (24). The maximal dose (tolerated dose) of free LDM for mice is 0.05 mg/kg. In the present study, LDM was linked with mlFVII to target TF for colon cancer therapy. The therapeutic dose of mlFVII-LDP-AE was increased to 0.8 mg/kg. The therapeutic efficacy was superior to that of LDM (0.05 mg/kg). Therefore, mlFVII-LDP-AE is an efficient cancer targeting therapeutic agent.

In conclusion, the present study reports for the first time that an mlFVII-targeted LDM effectively inhibited the growth and metastasis of mouse colon cancer. As human TF and FVII have features similar to those of mice, hlFVII-LDP-AE may be expected to have therapeutic potential for human colon cancers.
